# Keeping up with the times: how national public health and governmental organizations communicate about cannabis on Twitter

**DOI:** 10.1186/s13011-019-0224-3

**Published:** 2019-09-12

**Authors:** Jenna van Draanen, Tanvi Krishna, Christie Tsang, Sam Liu

**Affiliations:** 1 Faculty of Arts, Department of Sociology, University of British Columbia, Vancouver, BC Canada; 2 Faculty of Arts, Department of Psychology, University of British Columbia, Vancouver, BC Canada; 3 Faculty of Arts, School of Social Work, University of British Columbia, Vancouver, BC Canada; 4 Faculty of Education, School of Exercise Science, Physical & Health Education, University of Victoria, Victoria, BC Canada

**Keywords:** Cannabis, Marijuana, Messaging, Education, Public health, Social media

## Abstract

**Background:**

Public health and governmental organizations are expected to provide guidance to the public on emerging health issues in accessible formats. It is, therefore, important to examine how such organizations are discussing cannabis online and the information that is being provided to the public about this increasingly legal and available substance.

**Methods:**

This paper presents a concise thematic analysis of both the volume and content of cannabis-related health information from selected (*n* = 13) national-level public health and governmental organizations in Canada and the U.S. on Twitter.

**Results:**

There were eight themes identified in Tweets including 1) health-related topics; 2) legalization and legislation; 3) research on cannabis; 4) special populations; 5) driving and cannabis; 6) population issues; 7) medical cannabis, and 8) public health issues. The majority of cannabis-related Tweets from the organizations studied came from relatively few organizations and there were substantial differences between the topics covered by U.S. and Canadian organizations. The organizations studied provided limited information regarding how to use cannabis in ways that will minimize health-related harms.

**Conclusions:**

Authoritative organizations that deal with public health may consider designing timely social media communications with emerging cannabis-related information, to benefit a general public otherwise exposed to primarily pro-cannabis content on Twitter.

## Background

Although the platform is just over 10 years old, Twitter has significantly changed the way we interact with media. Twitter allows users to produce and consume information in 280-character segments (revised from 140 characters in 2017); with posts ranging from updates on important current events to mundane personal musings, it has changed the way in which we intake all types of information and given us the ability to connect with others instantaneously. These messages, or Tweets, have been used to analyze public opinion on a range of health topics. [1–4]

The public health community uses Twitter occasionally for the spread of health information. [5, 6] Advantages of spreading public health information this way include limited costs, wide audiences, and timely information exchange, but these come at the cost of perceived lack of credibility and difficulty for the audience in distinguishing opinion from evidence. [6] Paul and Dredze studied state public health department adoption of social media and found that 60 % of public health departments in the United States (U.S.) were using at least one type of social media; among these, 86.7% had a Twitter account [5]. Public health departments had an average of 983 Twitter followers, although there was considerable variety in the reach of messages, depending on the organization [5]. On average, state public health departments tended to post on social media only once per day, and substance use was not among the topics commonly posted [5].

In both Canada and the U.S., there has been considerable policy change recently, regarding the decriminalization and even legalization of cannabis. Several U.S. states have passed legislation to legalize recreational use of cannabis (e.g., Colorado, Washington, California) and the federal government in Canada has done the same. The continental landscape of cannabis policy is shifting, as is cannabis discussion online. Thompson and colleagues assessed cannabis-related Tweets posted by adolescents in 2012 and found that a majority (65.6%) of Tweets by adolescents reflected a positive attitude toward cannabis, and 42.9% indicated personal use. [7] Both Tweets about personal cannabis use and positive perceptions about cannabis increased from 2012 to 2013. [7] Cavazos-Rehg and colleagues analyzed the sentiment of a random sample of Tweets related to cannabis in 2014 and, again, found that most had a positive sentiment towards use, with pro-cannabis Tweets outnumbering anti-cannabis Tweets by a factor of 15. [8]

Public health and governmental organizations are expected to provide guidance to the public on emerging health issues, even in the absence of perfect information about risks and benefits. It is, therefore, important to examine how such organizations are discussing cannabis online and the information that is being provided to individuals who need to navigate safe use or decisions related to non-use of this increasingly legal and available substance. This paper provides a concise analysis of both the volume and content of cannabis-related information from selected public health organizations in Canada and the U.S. on Twitter.

## Methods

### Sampling and data collection

A total of *n* = 41,600 Tweets were collected in September 2017 using the Twitter application programming interface (API), were stored in CSV files, and were analyzed using Dedoose Software. [9] Public health and mental health/substance use organizations in Canada and the U.S. were eligible for inclusion in the analysis if they met the following criteria: had an active Twitter account (posted at least once per month in the previous 12-month period), provided national services or had a national scope/mandate of their work, were either a governmental organization or a not-for-profit organization, and posted a Tweet at least once in the last 12 month period about mental health or substance use issues.

The screening criteria yielded the following organizations in Canada for inclusion in our dataset: The Public Health Agency of Canada (PHAC) @PHAC_GC, Health Canada (HC) @GovCanHealth, the Canadian Public Health Association (CPHA) @CPHA_ACSP , The Canadian Mental Health Association (CMHA) @CMHA_NTL, the Mental Health Commission of Canada (MHCC) @MHCC_, the Canadian Institutes of Health Research (CIHR) @CIHR_IRSC, and the Centre for Addiction and Mental Health (CAMH) @CAMHNews; and the following organizations in the U.S.: The Department of Health and Human Services (DHHS) @HHSGov, The National Institute on Drug Abuse (NIDA) @NIDAnews, The Centres for Disease Control (CDC) @CDCgov, the Substance Abuse and Mental Health Services Administration (SAMHSA) @samhsagov, the American Public Health Association (APHA) @PublicHealth, the National Alliance on Mental Illness (NAMI) @NAMICommunicate, and the National Institutes of Health (NIH) @NIH.

This study is not intended to provide a comprehensive inventory of all public health organization activity on Twitter. Instead, key national organizations (*n* = 13) were selected with the intent of offering descriptive information about the volume and content of their public health messaging related to cannabis consumption. A total of 3200 of the most recent Tweets per organization (resulting in a total sample of 41,600 Tweets) were selected to be included in the initial dataset. For some organizations, based on the frequency of their Tweets, this meant that the period of analysis went back as far as 2011, for others it only went back to 2015. Keyword searches were conducted on this dataset to retain only the Tweets relevant to cannabis that contained any of the terms: ‘weed,’ ‘THC,’ ‘cannabis,’ ‘marijuana,’ ‘medical marijuana,’ or ‘pot,’ yielding the intermediate datasets depicted in Table 1. From this pool, re-Tweets were excluded and researchers hand-sorted Tweets to identify the ones relevant for analysis, producing the final datasets depicted in Table 1. The data used in this study are public and as such this study is exempt from human subject’s review.

**Table 1:** Screening and Resulting Sample Size of Cannabis-related Datasert for Included Organizations

	Canadian Organizations							U.S. Organizations						
Organization Name	CPHA	CAMH	HC	CMHA	PHAC	MHCC	CIHR	NIDA	SAMHSA	NIH	APHA	NAMI	CDC	DHHS
Starting dataset (n)	3200	3200	3200	3200	3200	3200	3200	3200	3200	3200	3200	3200	3200	3200
	↓	↓	↓	↓	↓	↓	↓	↓	↓	↓	↓	↓	↓	↓
Dataset after keyword filter (n)	200	158	81	50	117	143	87	345	114	120	17	55	134	276
	↓	↓	↓	↓	↓	↓	↓	↓	↓	↓	↓	↓	↓	↓
Dataset after RT removal and irrelevant Tweets removed (n)	108	84	47	5	4	1	5	291	14	3	17	0	0	1

### Data analysis

Data were analyzed using thematic content analysis. First, two researchers open-coded a selection of 50 Tweets and generated a codebook to be used in the first round of focused coding. Next, during focused coding, each researcher coded 100 Tweets according to these initial codes. After this initial focused coding, the researchers met to discuss and refine the themes according to emerging sub-themes. This revised codebook was then used to classify all Tweets, with each Tweet categorized by two researchers, after which the researchers again met to resolve disagreements in coding. Tweets were allowed to be classified in more than one thematic area where appropriate. Inter-rater reliability was calculated using a random sample of 100 Tweets, using Dedoose software [9] and the pooled Kappa statistic was found to be 0.94 [10] indicating excellent reliability. [11]

## Results

The majority of cannabis-related Tweets from the organizations studied come from relatively few organizations: CAMH (84/3200), CPHA (108/3200), and NIDA (291/3200). See Table 2 for a summary of the volume of Tweets, date range of included data, number of followers for each included organization, and the thematic content of their cannabis-related Tweets. The organizations with the most followers were the CDC (863,534), the NIH (811,577), and the DHHS (711,590); notably, they had few (4) cannabis-related Tweets combined. Tweets from the included health organizations were primarily information-sharing Tweets with links to research results, guidelines, policies, or fact sheets.

**Table 2:** Volume of Cannabis-related Tweets, Date Range Studied, Number of Followers, and Frequency of Themes in Tweets from Included Public Health and Governmental Organizations

Public Health and Governmental Organizations															
Twitter Characteristics	Canadian Organizations							United States Organizations							
	CPHA	CAMH	HC	CMHA	PHAC	MHCC	CIHR	NIDA	SAMHSA	NIH	APHA	NAMI	CDC	DHHS	
Number of followers*	3902	39,802	215,021	21,364	73,964	18,552	41,169	40,374	75,636	811,577	476,891	106,504	863,534	711,590	
Date range of included data (mm/yy)	02/15–09/17	07/16–09/17	08/13–09/17	07/12–09/17	11/11–09/17	05/15–09/17	12/15–09/17	04/15–09/17	01/16–09/17	06/15–09/17	08/16–09/17	02/16–09/17	08/16–09/17	05/16–09/17	
Number of cannabis-related Tweets (/3200)	108	84	47	5	4	1	5	291	14	3	17	0	0	1	*N* = 580
Theme/ Sub-theme Frequency ┼														Total n (%)	
T1: Health-related topics														103 (17.8%)	
Therapeutic effects	2							20							22
Smoke (cannabis & tobacco)		3						3							6
Neurological impacts		10		1				37		1					49
Substance use disorders								10							10
Cannabis and mental illness		5		2				9							16
T2: Legalization and legislation														103 (17.8%)	
Expert discussions	6	10	3					3							22
Governmental announcements	5		8												13
Issues with legalization	12	1						6							19
Promoting legalization with regulation	13	3	7												23
Promoting proposed legislation	4		12												16
Advertising public consultation			8	1				1							10
T3: Research on cannabis														101 (17.4%)	
Cannabis and alcohol		2						21		1					24
Cannabis and opioids								8		1					9
Sharing research results	3	15		2			1	45			1				67
Removing barriers to research	1	2						7							10
T4: Special Populations														92 (15.9%)	
Parents								8							8
Pregnant women								9							9
Youth and young adults	5	4	3	1				47	6	1					67
College students								8							8
T5: Driving and cannabis	3	5						51						59 (10.2%)	
T6: Population-level issues														38 (6.6%)	
Public perception of cannabis		1						10	2						13
Cannabis use rates	3	1	1					11	4	1	4				25
T7: Medical cannabis	18	3	3					11						35 (6.0%)	
T8: Public health topics														22 (3.8%)	
Approach to regulation	9	1	1		3										14
Promoting low-risk guidelines	4	3						1							8
Totals	88	69	46	7	3		1	326	12	5	5				562

┼ Tweets that did not fall in any existing themes or subthemes were included in an “other” category not depicted here, coding more than one category per Tweet was allowed

*Number of followers as of Oct 19, 2017

There were a total of eight themes found in the coded dataset, listed and described in order of frequency below. Twenty-three subthemes were present within the main themes and have been identified with bold font in the theme descriptions below.

### T1: health-related topics

One hundred three Tweets (or close to 18% of the 580 Tweets in the dataset) contained themes related to human health, most commonly about the **neurological issues** or brain changes that can arise when using cannabis (49 Tweets). However, within this theme, subthemes of beneficial or **therapeutic effects** of cannabis use, the impact of cannabis on the development of **mental illness** or effects of using cannabis for people with psychiatric disorders, the relationship between cannabis use and **substance use disorders**, and lastly **smoking** (tobacco and cannabis) were also found. Examples of health-related Tweets about neurological issues include the National Institutes of Health Tweet, “*Cannabis can be addictive & has negative effects on attention, memory, & learning:* *https*://*t.c* *#MTF2015”*and the Centre for Addiction and Mental Health Tweet*, “Quitting cannabis use improves #cognition in people with schizophrenia.”* Notably, as Tweets were allowed to be coded into more than one category, the CAMH Tweet example above was also coded in the cannabis and mental illness sub-theme. Health-related topics were Tweeted about relatively more frequently by U.S. organizations than Canadian organizations.

### T2: legalization and legislation

Nearly one in five Tweets analyzed (103 Tweets, or 18%) were related to legalization of cannabis and/or cannabis-related legislation. Most frequently (23 Tweets), the social media activity was **promoting legalization** along with careful regulation of the substance; or (22 Tweets) involved experts such as substance use researchers or policy makers offering **expert opinions** on legalization. Compared to U.S.-based organizations, Canadian health organizations Tweeted more frequently about legalization, regulation, and legislation, driving most of the content within this theme (as might be expected given recent federal policy changes in Canada). An example of a representative Tweet from this theme, from Health Canada is, “*Have your say on legalization & regulation of #cannabis* *https*://*t.c**…*” Other subthemes in this category included: discussions about the **issues with legalization** (for example, how to restrict access for minors); **promoting proposed legislation**, which usually involved discussion about a particular bill or law; **governmental announcements** about policy change; and advertising upcoming **public consultations** about cannabis regulation.

### T3: research on cannabis

Research on cannabis was highlighted and referenced in many posts (101 Tweets or 17%), for example, the National Institute on Drug Abuse shared a Tweet, *“What are cannabis’s long-term effects on the brain? To find out, see our updated Cannabis Research Report.* *https://t.c**.”* Most commonly, the post was generally designed to **publicize research results** (67 Tweets) and referenced a new paper, study, or report, without describing any direct application to consumers. Research also commonly highlighted interactions between **cannabis and alcohol** (24 Tweets) or **cannabis and opioids** (9 Tweets). A small number of Tweets described financial and legislative **barriers to research** and the way that legalization would make research more feasible. Highlighting research results via Twitter was done more commonly by U.S. than Canadian organizations.

### T4: special populations

Special populations were also mentioned frequently in Tweets (92 Tweets or 16%) usually with the Tweets discussing prevalence rates or unique considerations for cannabis use within particular populations, including **youth and young adults**, **pregnant women**, **parents**, and **college students**. Youth and young adults were by far the most commonly referenced population group, for example, SAMHSA Tweeted “*Acting Dir Baum: 20.8% of 18-25 year olds reported past month #cannabis use. Highest rate ever over past 15 years. #recoverymonth.”*

### T5: driving and cannabis

Just over 10% of the Tweets analyzed contained information about the effect that cannabis has on driving and warnings about driving while or after using cannabis. There was limited variation in the nearly 60 Tweets in this category, and as such there were no sub-themes. NIDA, a U.S. Organization, was responsible for the majority of Tweets in this category, Tweeting, for example, *“Dr. Marilyn Huestis discusses how #marijuana impairs a measure of driving.* *https://t.co/FC4mgq6j3w”*

### T6: population-level issues

Nearly 7% of the Tweets about cannabis by public health and governmental organizations were concerning issues at the population level, like **cannabis use rates** and public opinion or **perception of cannabis**. These population-wide topics came up more frequently in U.S. than Canadian organizations.

### T7: medical cannabis

Using cannabis for medicinal purposes, and Tweets that were about therapeutic use (vs. recreational use) or medical marijuana licensure or prescription were contained in a medical cannabis theme that included 6% of the total Tweets. This theme did not have sufficient variety to warrant subthemes. An example of a Tweet in this theme comes from CAMH, *“New research from CAMH: Medical marijuana programs and implications for cannabis control policy* *http://t.co/S0gyzvOxI3**”.*

### T8: public health topics

The least common theme, containing 22 Tweets, was public health topics. Content related to health outcomes and medical issues were captured in T1, and as such, most of the explicit public health content from the organizations concerned **public health approaches to regulation** and **sharing low-risk guidelines** for cannabis use to minimize harm. For example, the Public Health Agency of Canada Tweeted, “*#GoC committed to comprehensive #publichealth education on impacts of #cannabis.* *https://t.co/WdxDFyqH4P*”.

## Discussion

The organizations studied provided limited consumer-focused information regarding how to use cannabis in ways that will minimize health-related harms. Although the content of the Tweets did often relate to health issues (18% of Tweets), the nature of the Tweets in the dataset was primarily of a promotional or information-sharing style and rarely included specific advice, education, or instruction for followers. Given the pro-cannabis sentiment of much of the cannabis-related Tweets from the general public [12], Twitter users would benefit from more public-health driven content containing educational information related to health impacts of cannabis use. Some examples of educational messages designed to target the general public and to help maximize safety and health when using cannabis include, “start low and go slow” as well as, “use cannabis in a safe and familiar environment with people you trust” and, “if you are a new consumer, look for a product with less than 100 mg/g (10%) THC, with equal or higher levels of CBD,” etc. [13].

The use of cannabis for recreational purposes remains illegal federally in the United States, with some states adopting laws that permit legal recreational and/or medical use. As of July 2019, 11 states in the U.S. had passed laws permitting the use of cannabis for adults, whereas at the end of the period of data collection for this study there were 8 states where use was legal [14]. In Canada, a national approach to legalization was taken, with the use of cannabis for recreational purposes becoming legal on October 17, 2018 [15]. These different approaches to legalization undoubtedly affect how the organizations in our sample communicate with the public about cannabis use.

While health-related topics, population-level issues, and Tweets about research on cannabis were more common in the U.S. organizations studied, Tweeting about legislation, legalization, and regulation were more common in the Canadian organizations. It is possible that the impending policy change to legalize cannabis in Canada (at the time period studied) offered a unique policy window where non-governmental organizations could propose benefits of legalization and approaches to regulation that would be considered by policy makers, and where governmental organizations could advertise consultations and public dialogue about cannabis without fear of promoting criminalized activity, making discussion about legislative changes both more timely and relevant. In the absence of legislative change at the national level or indications of consideration of a national policy change, the U.S. organizations studied may have opted to Tweet information about other topics more salient to their followers and less politically objectionable. When approaching legalization, though, health-related topics become increasingly relevant to the general public and the relatively lower frequency of posting about health outcomes in Canadian organizations may represent a critical absence of useful information for Canadian Twitter users. Further, legalization is in itself a public health issue, and one that non-governmental organizations could be advancing discussion of, even without any actual or impending policy decisions from government. 

Based on our findings, it appears that in both countries public health and governmental organizations are not capitalizing on social media’s interactive potential and could be providing more direct information that is related to what Twitter users are discussing. [7] The organizations included in our study rarely posted about cannabis on Twitter, with the exception of NIDA, CAMH, CPHA, and HC and when they did post cannabis-related content it was primarily sharing research findings, reports, or links that contain information such as issues for youth and young adults, driving and cannabis use, neurological impacts of cannabis use, and medical cannabis.

There are other substances that have a heavily imbalanced “pro use” presence on social media, such as alcohol [16, 17], and this warrants further discussion as the public may benefit from public health and mental health/substance use organizations offering more information about how to use a variety of substances in ways that maximize health and safety, not just cannabis. Future research investigating other substance use-related content from health organizations may be needed. In addition, investigating how social media strategy is crafted and to what aim within such organizations is warranted.

Paul and Dredze report findings about the interactions between public health departments and Twitter users. [5] The departments in their study posted on average once per day and re-Tweets constituted 22.5% of all their Tweets; only 1.5% of Tweets were in response to a Tweet made by a follower. [5] This one-way social media communication pattern represents a missed opportunity to engage with Twitter users about information they may be seeking. Public health and governmental organizations would be well advised to incorporate social media into their substance use communication strategy, and given the recent switch to allow up to 280 characters on Twitter, this may be more feasible than it was previously. [5, 18]

### Limitations

Health organizations were selected by searching the internet for representative organizations that met search criteria and by identifying an institutional account from each organization’s web page. It is possible that individual programs or units within an organization are also using Twitter and were not identified. It is also possible that other public health agencies and non-governmental organizations not included in the sample were Tweeting relevant content that was not captured. Thus, the results of this analysis should not be extrapolated to institutions beyond the scope of this study. Future research should be designed to examine state or provincial Twitter activity to better understand regional differences in public health education messages about cannabis on Twitter. Moreover, this analysis does not allow us to comment on cannabis conversation on other social media platforms or public health education campaigns that may occur outside of Twitter: another avenue worth exploring in future research.

## Conclusions

Public health and governmental organization are tentative or occasional Twitter users with respect to using Twitter as a venue for communicating cannabis-related health information. As social media is becoming ever more pervasive, these organizations should consider ways to use Twitter to engage their audiences and create relationships which are usually hindered by budget and geographic restrictions. Given the pro-cannabis sentiment of the cannabis-related Tweets from the general public, Twitter users would benefit from more targeted public health-driven content containing educational information related to health impacts of cannabis use.

## Data Availability

The datasets generated and/or analysed during the current study are available in the Twitter API repository, https://developer.twitter.com/

## Abbreviations

APHA: American Public Health Association
CAMH: Centre for Addiction and Mental Health
CBD: Cannabidiol
CDC: Centres for Disease Control
CMHA: Canadian Mental Health Association
CPHA: Canadian Public Health Association
HC: Health Canada
DHHS: Department of Health and Human Services
MHCC: Mental Health Commission of Canada
NAMI: National Alliance on Mental Illness
NIDA: National Institute on Drug Abuse
NIH: National Institutes of Health
PHAC: Public Health Agency of Canada
SAMHSA: Substance Abuse and Mental Health Services Administration
THC: Tetrahydrocannabinol
U.S.: United States
